# A Case of Typhoid Fever with a Peculiar Complication

**Published:** 1882-07

**Authors:** J. H. Pryor

**Affiliations:** Resident Physician General Hospital


### A Case of Typhoid Fever with a Peculiar Complication.

Reported by J. H. Pryor, M. D., Resident Physician General Hospital.

L. L., aet. 36, single, German, laborer, entered General Hospital April 27, 1882. Presents appearance of one suffering from a fever. Upon question he stated that he had been sick for one week, and then followed a recital of the symptoms of typhoid fever. He was strong and of inferior intellect. Pulse 100, temperature 103, respiration 23. The treatment was simple, consisting of sponge baths, hydrochloric acid, m iii every 6 hours, and milk diet. No unusual symptoms presented themselves until the morning of May 23d, when it was noticed that he was drowsy and stupid, and complained of feeling very weak. He was ordered whiskey ℥ss every 3 hours. At 3 o'clock, P. M., same day, he had a profuse hæmorrhage from the bowels. The pulse immediately became weak, fluttering and rapid. A clammy sweat covered his body. Whiskey increased to ℥i every hour, and ergot in ʒss doses injected under the skin. He soon became unconscious, would not rally, and died at 3 o'clock, P. M., May 24, 1882. Autopsy same day.

No peritonitis. Ileum and ascending colon showed external sign of inflammation. Deep ulcerations in ascending colon; also ulceration of Peyer's patches for a considerable distance in the ileum. Cœcum dark colored and somewhat gangrenous. Pelvic cavity full of fœces. Lower half of rectum gangrenous, greatly softened and broken down. Large abscess located in its peritoneal fold. Searching carefully, found a large perforation in the rectum through which fœces were oozing. Surrounding tissues including a small portion of the bladder implicating in the diseased process. Attention was then called to the left leg, it being greatly swollen and emphysematous. The thigh gave token of being distended with fluid, the leg with gas. The origin of the fluid and the gas became a matter of interesting inquiry. By pressing upon the thigh fluid and bubbles of gas were forced to escape into the pelvic cavity. Finding the opening indicated, a cathether was introduced and allowed to trace its way into the thigh. Its point stopped just below the fold of the nates where it could be felt superficially. A careful dissection now revealed, that the fœces and fœcal gases after escaping from the rectum, had made their way, with the aid of ulceration and gangrene, through the pelvic fascia, escaping from the pelvis between the pyriformis and gemelli muscles and burrowed right through the glutei muscles.

The cellular tissue was infiltrated and its meshes distended clear to the ankle. Wherever the fluid fœces touched the muscles gangrene had occurred. So greatly was the leg distended by the gases that the skin had the feeling of a drum-head, and tapping it brought forth the same sound. The fatal hæmorrhage occurred because ulceration laid open one of the hæmorrhoidal arteries and let the blood escape. Many physicians at the autopsy pronounced the case (so far they knew) unique. Its recital should give rise to many questions. A glance at the chart shows a plain history of a light case of typhoid with every indication of convalescence during the last week. The symptoms are especially well clustered. Is it not strange that this grave complication proceeded to its terminate without an outward sign or manifestation.

